# Prognostic factors of disability progression in multiple sclerosis in real life: the OFSEP-high definition (OFSEP-HD) prospective cohort in France

**DOI:** 10.1136/bmjopen-2024-094688

**Published:** 2025-04-07

**Authors:** Francis Guillemin, Romain Casey, Jonathan Epstein, Yohann Foucher, David Laplaud, Hamza Achit, Fabien Rollot, Emmanuelle Leray, Sandra Vukusic, OFSEP-HD investigators, Eric BERGER

**Affiliations:** 1 CHRU, INSERM, Université de Lorraine, CIC Clinical Epidemiology, Nancy, Grand Est, France; 2 Université de Lorraine, INSERM, INSPIIRE, Paris, Île-de-France, France; 3 Université de Lyon, Université Claude Bernard Lyon 1, Lyon, France; 4 CIC 1402 INSERM, CHU de Poitiers, Université de Poitiers, Poitiers, France; 5 CHU Nantes, Service de Neurologie, CRC-SEP, Nantes Université, INSERM, CIC 1413, Center for Research in Transplantation and Translational Immunology, UMR 1064, F-44000, Nantes, France; 6 Univ Rennes, EHESP, CNRS, INSERM, Arènes—UMR 6051, RSMS (Recherche sur les Services et Management en Santé)—U 1309, Rennes, France

**Keywords:** Multiple sclerosis, Prognosis, Disabled Persons, Patient Reported Outcome Measures

## Abstract

**Abstract:**

**Purpose:**

To determine prognostic factors of disability in multiple sclerosis (MS), that is, (1) identify determinants of the dynamics of disability progression; (2) study the effectiveness of disease-modifying treatments (DMTs); (3) merge determinants and DMTs for creating patient-centred prognostic tools and (4) conduct an economic analysis.

**Participants:**

Individuals registered in the French Observatoire Français de la Sclérose en Plaques (OFSEP) database were included in this OFSEP-high definition cohort if they had a diagnosis of MS, were ≥15 years old and had an Expanded Disability Status Scale (EDSS) score <7. The outcomes will be assessed annually: (1) time to reach irreversible EDSS scores of 4, 6 and 7; (2) relapses and disease progression; (3) MRI-based progression, patient-reported outcomes, social consequences; and (4) combined outcomes on activity and progression. Clinical and quality-of-life data, MRI results and biological (blood, serum) samples will be collected at each follow-up.

**Findings to date:**

A cohort of 2842 individuals, 73.4% women, mean (SD) age of 42.7 (11.6) years, median disease duration of 8.8 years, has been recruited from July 2018 to September 2020. The course of MS was relapsing remitting in 67.7%, secondary progressive in 11.9%. The mean annual relapse rate was 0.98. The disease-modifying treatment received was highly effective therapy in 50.3% and moderately effective therapy in 30.7%.

**Future plans:**

The participants will be followed until December 2026. Disease course up to four landmarks will be examined as predictors of disease progression: (1) diagnosis of MS; (2) relapse activity worsening and independent progression; (3) any recent disease activity and (4) any visit with absence of disease activity in the past 5 years. The marginal effectiveness and tolerability of treatments will be assessed. Stratified algorithms will be proposed for medical decision-making. Economic evaluation of disease cost and cost-effectiveness of new DMTs will be conducted from a public payer perspective.

**Trial registration number:**

NCT03603457.

**STRENGTHS AND LIMITATIONS OF THIS STUDY**

- This cohort will be unique and large enough for comprehensive analysis integrating multiple potential determinants of multiple sclerosis (MS) prognosis, including sociodemographic, clinical, imaging data and treatments.
- Multimodal disability including clinical, imaging and patient-reported outcomes will be the target for prediction from specific landmarks, corresponding to strategic times in MS evolution.
- The collection of health-related quality of life will constitute a major advantage to evaluate the usefulness of prognostic tools in stratified medicine.
- Statistical analysis including specific landmarks integrated into dynamic modelling will allow for developing accurate prognostic analysis over time.
- A maximum follow-up no longer than 8 years will be a limitation to the prediction.

## Introduction

 Multiple sclerosis (MS) is a chronic disease affecting the central nervous system. It is the most common cause of non-traumatic neurological disability in young adults. With a mean age of diagnosis of 32 years and a 2:1 ratio of women to men, about 2.9 million persons worldwide are affected^1^ and nearly 2 in 1000 individuals in France in 2021.^2^ MS leads to permanent disability for decades, with marginal effect on life expectancy.^3^ The burden of MS is huge for societies, estimated at about 14.6 billion euros per year in Europe in 2010. It is rapidly increasing with the approval and wide use of expensive new disease-modifying therapies (DMTs),^4^ reaching an annual cost burden of 2.7 billion euros in France in 2020.^5^ However, disease progression remains difficult to treat even with the most recently approved drugs.

One major unmet need for MS patients is a sufficient knowledge of the factors associated with disease progression. Also, reliable predictive tools that could be applied at the individual level and at different key moments in the disease course (landmarks) are lacking. Despite many cohort-based studies helping to identify prognostic factors and sometimes propose prognostic scores,^6^,^8^ developing a tool accurate enough to predict the many dimensions of outcomes in MS faces several challenges that have not been addressed together. Mostly issued from disease onset (ie, inception cohorts) and from data collected during routine visits to the neurologist, tools to predict long-term prognosis are hampered by intermediate evolution events (eg, relapses over time) that may modify the prognosis. A recent Cochrane review,^9^ searching for prognostic models to be used any time after diagnosis for predicting future disease course, identified 75 models that were insufficiently validated to be recommended for clinical routine use. Of note, the reports did not describe prognostication at key clinical landmarks when the neurologist needs to decide on a change in management during the disease course.

However, the gain of knowledge about many factors, particularly the progress in cerebral and spinal-cord MS lesion imaging, the standardisation and/or new definitions of clinical assessments,^10^ the genetic background^11 12^ and, above all, the recent availability of an increasing number of DMTs,^8^ may considerably change the prognosis of the disease and the ability to predict its evolution. The evolution also depends on demographic, socioeconomic context (education, profession), environmental^13^ and behavioural (alcohol consumption,^13^ smoking^14^ and eating^15^) factors.

The aim of the present study is to develop a tool accounting for this multiplicity of factors, the use of DMTs and the disease events that may occur over time for predicting clinical, MRI and patient-reported outcomes (PROs) important for both clinicians and patients with MS. Such a multidimensional approach should be applicable at significant moments (landmarks) and requires the collection of many variables at inclusion and during the follow-up of a large cohort.

Few registries or cohorts used PROs, in particular health-related quality of life (QoL), as outcomes or even prognostic factors of MS progression.^16^,^18^ Measuring the perception of the disease evolution from the patient point of view using PROs is of importance in the context of personalised medicine. It can be used to identify points that could be improved in the patient’s point of view not considered by standard clinical evaluation. In addition, QoL can be used as a prognostic tool in other diseases.^19 20^ In the scope of health-economic analysis, the national and international health authorities recommend conducting cost-utility analysis whenever QoL is a major consequence of health interventions.^21 22^

To our knowledge, with the exception of the MS PATHS initiative,^17^ a cohort similar to Observatoire Français de la Sclérose en Plaques (OFSEP), though not organised with as accurate and structured measurement times as in OFSEP-high definition (OFSEP-HD), no other cohort study of MS patients has yet proposed the prospective, multicentric and standardised collection of such multisource and multimodal data to (1) describe the disease progression and identify its determinants (ie, socioeconomic and clinical characteristics, QoL, behavioural and environmental factors, MRI, DMT use and biologic samples); 2) develop patient-centred prognostic tools for the main landmarks of MS progression and to help in decision-making; 3) evaluate the effectiveness of DMTs by clinical trial emulations; and 4) assess the cost of MS disease and the cost-effectiveness of DMTs. According to the existing OFSEP initiative,^23^ the main innovative feature of the OFSEP-HD cohort is to propose for the first time a database for the national and international community of researchers in MS, from fundamental studies of biomarkers to projects in public health. In parallel with these objectives, relevant methodological challenges must be addressed to improve the quality of results.

## Cohort description

### Study design

#### The OFSEP-HD cohort

**Figure 1:**
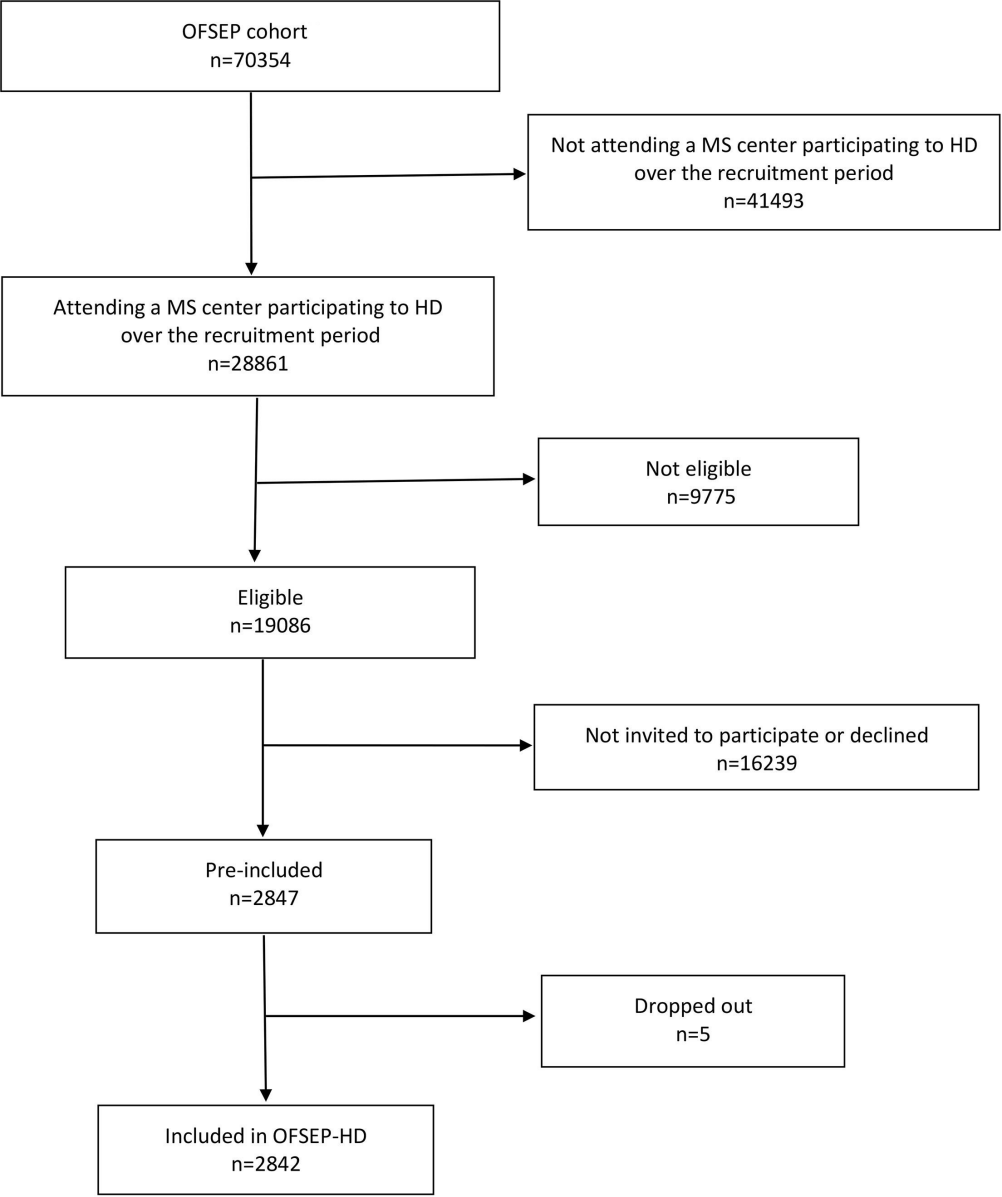
OFSEP-HD cohort recruitment flow diagram. HD, high definition; MS, multiple sclerosis; OFSEP, Observatoire Français de la Sclérose en Plaques.

The OFSEP-HD cohort is nested in the OFSEP cohort. Patient enrolment started on 10 July 2018 and ended on 11 September 2020 in 25 French MS centres. Individuals were eligible if they had (1) a diagnosis of MS according to the most recent criteria at entry into the HD cohort,^24^ (2) were ≥15 years old at inclusion, (3) had an MS diagnosis after the study start or, if MS onset occurred before the study start, had at least one visit every 2 years after follow-up in an MS centre with prospective OFSEP data collection; (4) had an irreversible Expanded Disability Status Scale (EDSS) score ≤7.0 (permanent use of a wheelchair) at inclusion in the study and (5) signed a written consent form. The criterion 3 for MS onset date allowed for mixing incident and prevalent cases, accelerating recruitment while benefiting from quality data collected during the OFSEP cohort follow-up, extending the possibility to fund the follow-up of included patients over time and increasing the probability to observe landmarks. Non-inclusion criteria were an inability to answer questionnaires and pregnancy at the time of inclusion (figure 1).

With a sample size of 2842 patients, a factor with a HR of 1.2 could be detected if the event rate was 30%, and one with a HR of 1.6 could be detected if the event rate was 5% (power=0.8; α risk=0.05; SD=0.5).^25^ We acknowledge that our power calculation does not account for multiple testing, as do other cohorts with many exploratory objectives.

This protocol is registered at ClinicalTrials.gov: NCT03603457.

#### The OFSEP cohort

The French OFSEP cohort is a nationwide systematic longitudinal study of individuals with MS followed in MS centres with more than 70 000 patient records collected in June 2018. The first objective was to provide a unique source of information on MS epidemiology, with a particular focus on pharmacoepidemiology of recently introduced DMTs. Since 2011, the centres have collected data with a standardised form as well as a minimal set of mandatory clinical data.^23^ They collect, organise and maintain the clinical database by using the MS-specific EDMUS software.^26^ In 2015, the OFSEP MRI working group published recommendations on the sequences to be used for regular brain and spinal cord MRI acquisitions of patients with MS,^27 28^ and standardised acquisition protocols have been disseminated. Pseudonymised MRI data are transferred to a centralised imaging resource centre, the Shanoir platform (http://shanoir.org). Moreover, biological samples are collected for a subsample of OFSEP patients and stored in a biobank.^29^

#### Multisource data collection

**Figure 2:**
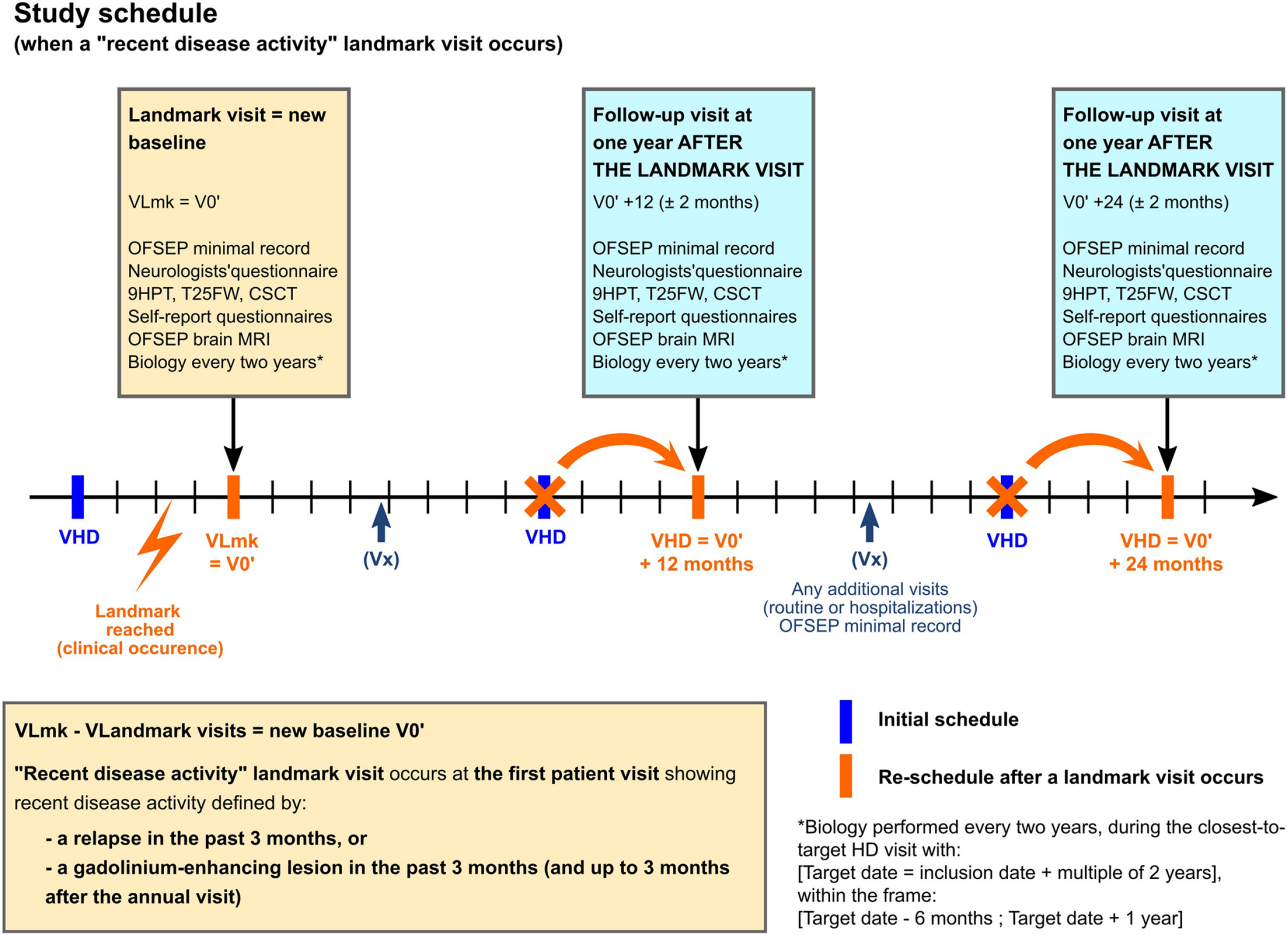
Design of the OFSEP-HD cohort. CSCT, Computerised Speed Cognitive Test; HD, high definition; 9HPT, 9-Hole Peg Test; OFSEP, Observatoire Français de la Sclérose en Plaques; T25FW, Time 25-Foot Walk.

During the inclusion visit in OFSEP-HD, historical clinical data were collected from medical records if they were incomplete from the OFSEP database, including oligoclonal bands and IgG index from cerebrospinal fluid at diagnosis. The OFSEP-HD follow-up visits are annual, with a time window of ±2 months around the anniversary inclusion date. In case of detection of recent disease activity (see landmarks paragraph below), a new baseline assessment at this key step of the disease (rebaseline) will be done, which will lead to restarting the annual follow-up (figure 2). The rebaseline process is allowed only once for a patient.

Four main landmarks are considered, corresponding to four strategic times in the MS evolution: (1) the first visit when the diagnosis of MS is set; (2) the first visit when the diagnosis of progression (primary or secondary) is set; (3) any visit with recent disease activity, defined by the occurrence of a relapse and/or MRI activity detected with gadolinium enhancement in the past 3 months (or gadolinium-enhanced lesion in the 3 months after an annual visit) and (4) any visit with absence of disease activity in the past 5 years.

At the inclusion and annual OFSEP-HD follow-up visits, the following multisource data are collected. First, the investigators oversee the collection of sociodemographic data, geographic residence, geographic area of origin, neurological episodes, EDSS, DMTs (date of onset and stopping, reasons for stopping), serious adverse events (OFSEP minimal report form), standardised brain and whenever possible spinal cord MRI results following OFSEP recommendations for MRI acquisitions,^27 28^ and additional clinical evaluation with Time 25-Foot Walk, 9-Hole Peg Test^30^ and Computerised Speed Cognitive Test.^31^ They also collect brain MRI results obtained in one of the labelled centres using the OFSEP acquisition protocols in a 3-month period before or after the inclusion visit and in a 2-month period before or after the follow-up visit. This time window is necessary to not undermine the routine follow-up of the patient, but special attention will be paid to organise the MRI examinations as close as possible to the visits. Biological samples (blood, serum) were collected at inclusion for constitution of a biobank^29^ and to dose (1) biomarkers (neurofilament light chain (NFL), Tau, Glial fibrillar acidic protein (GFAP), ubiquitin C terminal hydrolase L1) with Simoa Human Neurology 4-Plex assay and every 2 years with 2-Plex (NFL, GFAP) of Quanterix, which detects subpicogram levels of biomarkers, a very sensitive (limit of 0.32 pg/mL) and reproducible (coefficient of variation in the 10% range) method, (2) vitamin D with mass spectrometry, which is very sensitive and reproducible and (3) genetic ancestry. When necessary, we will use a part of the historical OFSEP biological collection. Establishing such a biological collection will allow to assess other markers (eg, EBV status) and help validate new serum biomarkers of MS in the future (eg, other neuronal or glial markers, cholesterol, oxidative stress, cytokine profile, auto-antibodies) as well as persistent organic pollutants possibly involved in the progression of MS.

Second, patient self-reporting questionnaires will be used to assess PROs (see below).

Third, the OFSEP-HD cohort will be linked to the French Système national des données de santé (SNDS) claims database registering all data for reimbursed health prescriptions and hospital stays covered by the national health insurance system.^2 32^ Sick leave and disability status/pension will be obtained from the SNDS as will use of healthcare (specific to and apart from MS), and geographic location with related socioeconomic variables and access to care.

### Outcomes

#### Clinical outcomes

These refer to physical disability commonly assessed with the EDSS^33^ and other parameters, such as activity and progression, as defined in the 2013 Lublin clinical classification.^34^ Clinical evaluations will be completed with more specific measures of physical and cognitive disability:

- Relapses defined as the occurrence, recurrence or worsening of symptoms of neurological dysfunction lasting>24 hour and usually ending with remission, partial or complete. Symptoms occurring within 1 month are considered part of the same relapse.
- Progression defined as the steady worsening of neurological symptoms and signs for at least 6 months, whether superimposed with relapses or not,^35^ including relapse activity worsening.

Disability is defined as irreversible when the assignment to a given score has been reached and persists for at least 6 months, excluding any transient worsening of disability related to relapses.^36^

#### MRI outcomes

These data represent surrogate markers of disease activity and progression. MRI annual acquisitions will conform to OFSEP recommendations for MRI standardised acquisitions in France.^27 28^ Because raw acquisitions will be available (3D FLAIR, 3D T1, diffusion images), many MRI measures will be assessable, in particular:

- T2/FLAIR image lesion load and identification of new lesions compared with previous MRI acquisitions, with measurement of their volume and number.
- Brain volume and atrophy using T1 images.

#### Patient-reported outcomes

These include the following questionnaires:

- Health-related QoL measured with the Medical Outcomes Study 12-items Short Form (SF-12), a self-reporting questionnaire based on the generic QoL SF-36 questionnaire.^37^ The SF-12 was scored with item response theory weights (RAND-12 HSI) that provide physical, mental and global scores.^38^
- The MusiQoL questionnaire related to specific MS characteristics.^39^
- Self-perceived health states with the EQ-5D 5L. These health states are associated with utility weights that can be used in an econometric approach.^40 41^
- Social consequences assessed by employment situation, sick leave occurrence and duration, unemployment or dependency. Employment situation will be declared by patients with MS at annual visits.

#### Combined outcomes

By considering both clinical and MRI data, combined outcomes allow for stratifying patients into active and progressive groups as defined by the last Lublin classification,^34^ in activity defined on clinical and/or MRI features and progression on clinical features only:

- No evidence of disease activity (NEDA) is a composite of measures related to disease activity and progression. It is derived from the post hoc analyses of contemporary phase 3 clinical trials of, for example, natalizumab and cladribine.^42^,^44^
- The Rio score^45^ and the modified Rio score^46^ combine new T2 image lesions and relapses.
- Progression independent of relapse activity will be also considered.^10^

#### Potential prognostic factors

Factors with a potential prognostic value will be studied in two different sets. First, specific attention will be paid to sociodemographic and clinical determinants and biomarkers of the progression of disability. Such characteristics include age, sex, level of education, occupation, residency, initial relapse, clinical MS form (primary progressive, relapsing remitting and secondary progressive), past and current disease activity, DMTs and comorbidities including health behaviours (smoking, alcohol, body mass index, etc). Comorbidities are assessed using the Functional Comorbidity Index^47^ and the Charlson Comorbidity Index adapted for claims databases.^48^ To enrich prognostic models with potential predictors of disease evolution, questionnaires will be annually proposed.

Second, high efficacy classes of DMT and switches as well as off-label prescriptions are available since disease onset. Nine DMTs are currently available for relapsing remitting MS and secondary progressive MS with activity. These drugs include interferons, glatiramer acetate, teriflunomide, sphingosine 1-phosphate receptor modulators, fumarates, cladribine and five types of monoclonal antibodies.^8 49^

#### Findings to date

A cohort of 2842 individuals has been recruited from July 2018 to September 2020. Their characteristics are described in online supplemental table S1). They are 73.4% women, with mean (SD) age at inclusion 42.7 (11.6) years. Their mean age at onset was 31.7 (10.2) years and median disease duration was 8.8 (4.3–15.7) years. At inclusion, the mean EDSS was 2.4 (1.9) and the course of MS was a unique episode in 14.4%, relapsing remitting in 67.7%, secondary progressive in 11.9% and primary progressive in 6.0%. The mean annual relapse rate was 0.98 (0.80–1.19). The disease-modifying treatment received was highly effective therapy in 50.3%, moderately effective therapy in 30.7%, while 6.4% were naive of DMT and 12.5% had no current treatment. Landmarks at inclusion were documented by an MS diagnosis for less than 6 months in 4.4%, MS progression in the past 12 months in 1.2%, relapse or MRI activity in the past 3 months in 25,5% (with 369 missing values) and remission (NEDA 3) in the past 5 years in 10.7% (with 34 missing values).

We compared the characteristics of 2847 included and 16 239 non-included individuals among 19 806 eligible MS individuals, according to inclusion criteria, attending the 25 centres over the period. The data are presented in online supplemental table S2 and show that among eligible MS individuals, those included have less MRI activity in the past 3 months, an MS diagnosis for more than 6 months, a moderate EDSS and receive more highly effective therapy, with a significant heterogeneity of recruitment across MS centres.

A comparison of their characteristics at inclusion showed that individuals with missing data on clinical outcomes (22.4%), PROs (13.5%) and landmarks (19.2%) were older, with MS onset or MS diagnosed at older age, had slightly lower education, more progressive phenotype, higher EDSS score, and received less active treatment than those without missing data (online supplemental tables S3–S5).

### Future plans

The study participants will be followed until 8 December 2026.

#### Statistical analysis strategy

Several prognostic models will be developed and validated. The model development will be based on a training cohort consisting of a random selection of two-thirds of the centres. The above list of prognostic factors (ie, first, sociodemographic, clinical, imaging, biological variables and comorbidities and second, treatments and the four landmarks) will be considered as three blocks. The prognostic factors will be considered to achieve dynamic predictions^50^ of the following times to events: reaching irreversible EDSS scores of 4, 6 and 7; first relapse; disease progression; MRI outcomes; PROs (QoL), social consequences and combined outcomes of clinical and MRI data reflecting activity and disease progression.

To consider the longitudinal predictors up to the landmark time, we will adapt the methodology proposed by Devaux *et al*.^51^

According to data from the remaining one-third of centres in the external validation cohort, we will use several metrics to appraise the predictive capacities of the models. We will randomise centres and not individuals for learning and validation, because the latter strategy would have been considered as internal validation, questioning the generalisation of the results.^52^

In a stratified medicine perspective, we aim to develop stratified algorithms for medical decision-making for maximising the number of years without disease progression and with the best QoL, this last dimension depending on both the disease activity and the treatment adverse events. The knowledge gained will allow for a clear picture of the history of MS in the 2010–2020s and the various determinants of outcomes. This landmark approach will identify some prognostic factors that play a permanent role and others to be considered at some stages of the disease course for more appropriate decision-making for care, regardless of treatment. We will study the potential of the new classification of disease activity and progression to modify the prognostic classification of cases initially developed using the classical progressive relapse secondary progressive classification.

#### Economic analysis plan

Two types of economic analysis will be conducted: (1) a cost-of-illness analysis for identifying the current cost burden to society, together with the identification of the main cost drivers and (2) a cost-effectiveness analysis for exploring the efficiency of recent DMT treatments, namely biotherapies, compared with standard treatments. The effectiveness of new treatments for previously defined outcomes will be assessed after controlling for the prognostic information determined above.

We expect the findings of this research from the OFSEP-HD cohort to have an impact on MS diagnosed adult, with limited disability progression and preserved ambulatory ability, whatever the disease duration, potentially eligible to DMT, and followed in a French expert centre, so representing a large proportion of French MS ambulatory people.

#### Collaboration

Scientific collaboration based on data sharing with other teams in the scientific community will be open and encouraged, in line with the funding body policy. Any scientific project will be examined by the OFSEP scientific committee, also in consideration of technical feasibility and full respect of general data protection rules.

### Patient and public involvement

Patient associations are already involved in different ways in the OFSEP project. To include patient representatives more formally in the governance of OFSEP, one patient representative from UNISEP, the national federation of MS patient associations, has joined the Steering Committee, with a voting right. Patients were not involved in the OFSEP-HD study research question or design.

### Ethics and dissemination

In accordance with French laws, ethical approval was obtained by a national institutional review board (ethical approval received on 15 June 2018 from the Comité de Protection des Personnes Sud-Ouest et Outre-Mer IV (no. CPP18-036a/2018-A00882-53)). Dissemination of the OFSEP purpose and objectives and dissemination of results will involve different directions and publics.

- The international scientific community through papers in peer-reviewed journals and abstracts in conferences.
- The French neurological community and participants of the OFSEP cohort.
- Patients and the public: this will be a good opportunity to give concrete examples of the impact of research, with direct impact on the management of patients. The involvement of a patient representative will allow for a better understanding of patient expectations and needs.

## Supplementary material

10.1136/bmjopen-2024-094688 online supplemental material 1

## Data Availability

Data are available upon reasonable request.
