# Supplementary material for: Prognostic factors of disability progression in multiple sclerosis in real life: the OFSEP-high definition (OFSEP-HD) prospective cohort in France
Source: BMJ Open. 2025 Apr 7;15(4):e094688. doi: 10.1136/bmjopen-2024-094688 (PMC12001352; doi:10.1136/bmjopen-2024-094688)
Supplement: online supplemental material 1 [file bmjopen-15-4-s001.docx]

**Table S1. Characteristics of the MS subjects at inclusion in the OFSEP-HD cohort (n=2842)***

|  | Number | | % |  |
| --- | --- | --- | --- | --- |
| ***Socio-demographic*** |  | |  |  |
| Age at inclusion (years)  mean (SD)  median (Q1-Q3) | 42.7 (11.6)  42.0 [34.0-51.0] | |  |  |
| Sex male  Female | 757  2085 | | *26.6*  *73.4* |  |
| Level of education university > 3yrs  university ≤ 3yrs  U college  *missing* | 529  843  1324  *146* | | *19.6*  *31.3*  *49.1* |  |
| Occupation employed  sick leave  retired  student  unemployed  *missing* | 1731  443 175 109  261  *123* | | 63.6  16.4  6.4  4.0  9.6 |  |
| ***Lifestyle*** |  | |  |  |
| Tobacco never smoker  former smoker  current smoker  *missing* | 1025  857  781  *179* | | 38.4  32.2  29.4 |  |
| Alcohol ≥ once a week  2-3 times a month  ≤ once a month  non consumer  never over lifetime  *missing* | 972  548  577  249  339  *157* | | 35.3  19.9  20.9  11.3  12.3 |  |
| ***Clinical*** |  | |  |  |
| Age at disease onset (years)  mean (SD)  median (Q1-Q3) | 31.7 (10.2)  30.4 [24.3-38.3] | |  |  |
| Age at diagnosis (years)  mean (SD)  median (Q1-Q3) | 35.0 (10.7)  34.1 \|27.0-42.1] | |  |  |
| MS phenotype unique episode  relapsing remitting  secondary progressive  primary progressive | 409  1925  337  171 | | 14.4  67.7  11.9  6.0 |  |
| EDSS (0-10)  mean (SD)  median (Q1-Q3) | 2.4 (1.9)  2.0 [1.0-4.0] | |  |  |
| Annual relapse rate in the past 2 years [CI 95] | 0.98 [0.80-1.19] |  |  |  |
| Comorbidity number**  mean (SD)  median (Q1-Q3)  Groll index  Charlson index | 0.46 (1.07)  0.0 (0-1)  1.82 (1.23)  0.46 (0.85) | |  |  |
| BMI (kg/m²)  mean (SD)  median (Q1-Q3) | 24.6 (5.1)  23.5 [21.0-27.1] | |  |  |
| ***Disease activity*** |  | |  |  |
| Episode in the past 3 months | 191 | | 6.7 |  |
| Episode in the past 12 months | 558 | | 19.6 |  |
| MRI activity in the past 3 months  *missing* | 561  *369* | | 19.7 |  |
| MRI activity in the past 12 months  *missing* | 951  *203* | | 33.5 |  |
| Remission (NEDA 3) in the past 12 months  *missing* | 850  *399* | | 29.9 |  |
| ***Landmarks at inclusion*** |  | |  |  |
| MS diagnosis for less than 6 months | 124 | | 4.4 |  |
| MS progression in the past 12 months | 38 | | 1.3 |  |
| Relapse or MRI activity in the past 3 months  *missing* | 631  *360* | | 25.4 |  |
| Remission (NEDA 3) in the past 5 years  *missing* | 305  *34* | | 10.7 |  |
| ***MS past monitoring*** |  | |  |  |
| Brain MRI density (nbr per year) [CI 95] | 0.54 [0.41-0.70] |  |  |  |
| Spinal cord MRI density (nbr per year) [CI 95] | 0.23 [0.15-0.34] |  |  |  |
| ***Disease modifying treatment*** |  | |  |  |
| Naive  No treatment | 182  355 | | 6.4  12.5 |  |
| Highly effective therapy | 1429 | | 50.3 |  |
| Moderately effective therapy | 872 | | 30.7 |  |
| Off label therapy or clinical trial | 4 | | 0.1 |  |
| ***Quality of life*** |  | |  |  |
| SF12*** physical component (0-100)  *missing*  mental component (0-100)  *missing* | 59.7 (27.0)  *242*  54.9 (31.4)  *242* | |  |  |
| Global score (0-100)  *missing* | 53.6 (22.5)  *242* | |  |  |
| MusiQol index (0-100)  *missing* | 70.5 (16.1)  *354* | |  |  |
| EQ-5D-5L (0-1)  *missing* | 0.867 (0.169)  *191* | |  |  |

* missing values are indicated where applicable; **reported by the neurologist;

Comparison of included (n=2847) vs not included (16239) among 19086 eligible MS individuals according to inclusion criteria

**Table S2. Adjusted odds ratios to be included in OFSEP-HD cohort among 19086 eligible MS individuals**

|  | **aOR*** | **95% CI** | **p-value** |
| --- | --- | --- | --- |
| **MRI activity at +/- 3 months from baseline** |  |  |  |
| Yes | 0.86 | 0.75-0.98 | 0.026 |
| No | 1 |  |  |
| Missing | 0.11 | 0.10-0.13 | <0.001 |
| **MS diagnosis for more than 6 months** |  |  |  |
| Yes | 0.29 | 0.24-0.37 | <0.001 |
| No | 1 |  |  |
| **EDSS (+/- 1 month)** |  |  |  |
| [0.0-3.5] | 1 |  |  |
| [4.0-5.5] | 0.91 | 0.79-1.03 | 0.145 |
| [6.0-7.0] | 0.69 | 0.58-0.81 | <0.001 |
| **Remission (NEDA 3) over the past 5 years** |  |  |  |
| Yes | 1 |  |  |
| No | 0.90 | 0.79-1.02 | 0.107 |
| Missing | 0.74 | 0.63-0.86 | <0.001 |
| **Disease modifying treatment at baseline** |  |  |  |
| Naïve | 0.26 | 0.21-0.31 | <0.001 |
| No treatment | 0.51 | 0.44-0.59 | <0.001 |
| Highly effective therapy | 1 |  |  |
| Moderate effective therapy | 0.58 | 0.52-0.64 | <0.001 |
| Off label therapy or clinical trial | 0.51 | 0.20-1.30 | 0.158 |
| **Time from MS onset to first clinic visit (yrs)** | 0.97 | 0.96-0.97 | <0.001 |

*Logistic regression model with center as a random effect (AIC= 11995), with significant likelihood ratio (p<0.001) compared to model with center as a fixed effect (AIC = 12680).

Comparing included (n=2847) vs not included (16239) among 19086 eligible MS individuals according to inclusion criteria, those included have less MRI activity in the past 3 months, a MS diagnosis for more than 6 months, a moderate EDSS, and receive more highly effective therapy, with a significant heterogeneity of recruitment across MS centers.

Comparison of patients with missing data vs no-missing data in the OFSEP-HD cohort

**Table S3. Baseline characteristics of patients with missing data on landmarks**

|  | **Missing data (n\|%)** | | **No missing data (n\|%)** | | **p value** |
| --- | --- | --- | --- | --- | --- |
|  |  |  |  |  |  |
| **Total** | **383** | **13.5** | **2459** | **86.5** |  |
| **Age at baseline**  Median [Q1-Q3] | 45.4 [36.3-54.7] | | 41.5 [33.7-50.4] | | <0.001 |
| **Age at MS onset**  Median [Q1-Q3] | 31.2 [24.0-40.0] | | 30.4 [24.3-37.9] | | 0.129 |
| **Age at MS diagnosis**  Median [Q1-Q3] | 35.2 [27.3-45.4] | | 34.0 [27.0-41.7] | | 0.016 |
| **Sex** |  |  |  |  | 0.805 |
| Men | 104 | 27.1 | 653 | 26.6 |  |
| Women | 279 | 72.9 | 1806 | 73.4 |  |
| **Level of education** |  |  |  |  | 0.257 |
| University > 3yrs | 62 | 16.2 | 467 | 19.0 |  |
| University ≤ 3 yrs | 105 | 27.4 | 738 | 30.0 |  |
| College | 194 | 50.7 | 1130 | 46.0 |  |
| Missing | 22 | 5.7 | 124 | 5.0 |  |
| **MS phenotype** |  |  |  |  | <0.001 |
| Unique episode | 50 | 13.0 | 359 | 14.6 |  |
| Relapsing remitting | 225 | 58.8 | 1700 | 69.1 |  |
| Secondary progressive | 76 | 19.8 | 261 | 10.6 |  |
| Primary progressive | 32 | 8.4 | 139 | 5.7 |  |
| **EDSS**  Median [Q1-Q3] | 2.5 [1.5-4.0] | | 2.0 [1.0-4.0] | | <0.001 |
| **ARR* in the past 2 years [95%CI]** | 0.83 [0.77-0.90] | | 1.00 [0.97-1.03] | | <0.001** |
| **Comorbidity number**  Median [Q1-Q3] | 0 [0-1] | | 0 [0-1] | | 0.511 |
| **Disease Modifying treatment** |  |  |  |  | <0.001 |
| Naïve | 28 | 7.3 | 154 | 6.2 |  |
| No treatment | 70 | 18.3 | 285 | 11.6 |  |
| Highly effective therapy | 150 | 39.2 | 1279 | 52.0 |  |
| Moderate effective therapy | 135 | 35.2 | 737 | 30.0 |  |
| Off label therapy or clinical trial | 0 | 0.0 | 4 | 0.2 |  |

*ARR=annual relapse rate

**from negative binomial regression

**Table S4. Baseline characteristics of patients with missing data on clinical outcomes**

|  | **Missing data (n\|%)** | | **No missing data (n\|%)** | | **p value** |
| --- | --- | --- | --- | --- | --- |
|  |  |  |  |  |  |
| **Total** | **637** | **22.4** | **2205** | **77.6** |  |
| **Age at baseline**  Median [Q1-Q3] | 44.2 [36.3-54.2] | | 41.2 [33.5-50.0] | | <0.001 |
| **Age at MS onset**  Median [Q1-Q3] | 31.5 [24.5-39.5] | | 30.1 [24.2-37.8] | | 0.017 |
| **Age at MS diagnosis**  Median [Q1-Q3] | 35.5 [27.9-44.9] | | 33.7 [26.9-41.6] | | 0.001 |
| **Sex** |  |  |  |  | 0.035 |
| Men | 149 | 23.4 | 608 | 27.6 |  |
| Women | 488 | 76.6 | 1597 | 72.4 |  |
| **Level of education** |  |  |  |  | 0.049 |
| University > 3yrs | 103 | 16.2 | 426 | 19.3 |  |
| University ≤ 3 yrs | 175 | 27.5 | 668 | 30.3 |  |
| College | 320 | 50.2 | 1004 | 45.5 |  |
| Missing | 39 | 6.1 | 107 | 4.9 |  |
| **MS phenotype** |  |  |  |  | 0.492 |
| Unique episode | 85 | 13.3 | 324 | 14.7 |  |
| Relapsing remitting | 443 | 69.5 | 1482 | 67.2 |  |
| Secondary progressive | 77 | 12.1 | 260 | 11.8 |  |
| Primary progressive | 32 | 5.0 | 139 | 6.3 |  |
| **EDSS**  Median [Q1-Q3] | 2.0 [1.5-4.0] | | 2.0 [1.0-4.0] | | <0.001 |
| **ARR* in the past 2 years [95%CI]** | 0.93 [0.88-0.99] | | 0.99 [0.96-1.02] | | 0.053** |
| **Comorbidity number**  Median [Q1-Q3] | 0 [0-1] | | 0 [0-1] | | 0.017 |
| **Disease Modifying treatment** |  |  |  |  | 0.034 |
| Naïve | 41 | 6.4 | 141 | 6.4 |  |
| No treatment | 100 | 15.7 | 255 | 11.6 |  |
| Highly effective therapy | 296 | 46.5 | 1133 | 51.4 |  |
| Moderate effective therapy | 200 | 31.4 | 672 | 30.5 |  |
| Off label therapy or clinical trial | 0 | 0.0 | 4 | 0.2 |  |

*ARR=annual relapse rate

**from negative binomial regression

**Table S5. Baseline characteristics of patients with missing data on patient reported outcomes**

|  | **Missing data (n\|%)** | | **No missing data (n\|%)** | | **p value** |
| --- | --- | --- | --- | --- | --- |
|  |  |  |  |  |  |
| **Total** | **546** | **19.2** | **2296** | **80.8** |  |
| **Age at baseline**  Median [Q1-Q3] | 45.9 [36.7-54.9] | | 40.9 [33.6-49.8] | | <0.001 |
| **Age at MS onset**  Median [Q1-Q3] | 32.7 [25.2-41.5] | | 29.9 [24.0-37.3] | | <0.001 |
| **Age at MS diagnosis**  Median [Q1-Q3] | 37.2 [27.9-45.9] | | 33.4 [26.8-41.3] | | <0.001 |
| **Sex** |  |  |  |  | 0.415 |
| Men | 153 | 28.0 | 604 | 26.3 |  |
| Women | 393 | 72.0 | 1692 | 73.7 |  |
| **Level of education** |  |  |  |  | <0.001 |
| University > 3yrs | 66 | 12.1 | 463 | 20.2 |  |
| University ≤ 3 yrs | 123 | 22.5 | 720 | 31.4 |  |
| College | 262 | 48.0 | 1062 | 46.2 |  |
| Missing | 95 | 17.4 | 51 | 2.2 |  |
| **MS phenotype** |  |  |  |  | <0.001 |
| Unique episode | 53 | 9.7 | 356 | 15.5 |  |
| Relapsing remitting | 351 | 64.3 | 1574 | 68.6 |  |
| Secondary progressive | 84 | 15.4 | 253 | 11.0 |  |
| Primary progressive | 58 | 10.6 | 113 | 4.9 |  |
| **EDSS**  Median [Q1-Q3] | 2.5 [1.5-4.0] | | 2.0 [1.0-3.5] | | <0.001 |
| **ARR* in the past 2 years [95%CI]** | 0.93 [0.87-0.99] | | 0.99 [0.96-1.02] | | 0.084** |
| **Comorbidity number**  Median [Q1-Q3] | 0 [0-1] | | 0 [0-1] | | 0.471 |
| **Disease Modifying treatment** |  |  |  |  | 0.115 |
| Naïve | 46 | 8.4 | 136 | 5.9 |  |
| No treatment | 69 | 12.6 | 286 | 12.5 |  |
| Highly effective therapy | 280 | 51.3 | 1149 | 50.0 |  |
| Moderate effective therapy | 151 | 27.7 | 721 | 31.4 |  |
| Off label therapy or clinical trial | 0 | 0.0 | 4 | 0.2 |  |

*ARR=annual relapse rate

**from negative binomial regression
