# Supplementary material for: Prognostic factors of disability progression in multiple sclerosis in real life: the OFSEP-high definition (OFSEP-HD) prospective cohort in France
Source: BMJ Open. 2025 Apr 7;15(4):e094688. doi: 10.1136/bmjopen-2024-094688 (PMC12001352; doi:10.1136/bmjopen-2024-094688)
Supplement: Supplementary file 2 [file bmjopen-2024-094688.reviewer_comments.pdf]

PEER REVIEW HISTORY

BMJ Open publishes all reviews undertaken for accepted manuscripts. Reviewers are asked to complete a checklist review form and are provided with free text boxes to elaborate on their assessment. These free text comments are reproduced below.

ARTICLE DETAILS

Title (Provisional)

Cohort profile: prognostic factors of disability progression in multiple sclerosis in real life: the OFSEP-high definition (OFSEP-HD) prospective cohort in France

Authors

Guillemin, Francis; Casey, Romain; Epstein, Jonathan; FOUCHER, Yohann; David, Laplaud; ACHIT, Hamza; Rollot, Fabien; Leray, Emmanuelle; Vukusic, S; investigators, OFSEP-HD

VERSION 1 - REVIEW

**Reviewer**	**1**
**Name**	**El Sankari , Souraya**
**Affiliation**	**Department of Neurology, Cliniques Universitaires Saint-Luc**
**Date**	**30-Nov-2024**
**COI**	**None**

In this paper from the OFSEP group, the authors aim to collect broad multi parametric multi center determinants of progression in MS. The study design and objectives are provided. The studied parameters are specified with details. the manuscript is well written. A few questions / remarks for minor corrections:

1) Are authors intending to collect and use data from LP (OCB / free kappa light chains) for prognostic purposes ?

2) Combined to smoking, BMI, are vit D levels available in baseline evaluations of patients . the same for the EBV serological status

3) Please check the citations style in the bibliography section. Many errors to be corrected, and the style to be checked.

**Reviewer**	**2**
**Name**	**Briggs, Farren**
**Affiliation**	**University of Miami Miller School of Medicine**

**Date**	**30-Dec-2024**
**COI**	**None**

This is an overview of a cohort study of MS patients recruited from 28 MS centers in France. The authors present an initial demographic/clinical description of subjects.

There are several opportunities to improve the document:

Page 7 line 20: The citations 16-18 refer to reviews of self-management interventions and exercise on QOL. They do not refer to registries or cohort that have successfully incorporated PROs, and leveraged them as outcomes or predictors (in cross-sectional and longitudinal analyses) in numerous studies.

For example: https://doi.org/10.7224/1537-2073.2020-133, https://doi.org/10.1016/j.msard.2019.01.045, https://doi.org/10.3389/fneur.2020.00632, etc. Thoughtful inclusion of appropriate citations would be an improvement.

Page 7 Line 34. Please see https://doi.org/10.3389/fneur.2020.00632 - this ongoing study is very similar to what the authors describe in the opening of the paragraph.

Page 8 Line 10, is there any information on enrolment rate of eligible PwMS from the 28 MS centers? Are the eligible patients at these 28 MS centers representative of the broader French MS population (i.e. are these centers only in urban centers?) In review of the CONSORT diagram (fig 1) – are there any concerns with selection bias, i.e. are the 30K at non-participating centers similar to the 41K at participating centers? Are the 16K who declined/not invited similar to the 2847 who enrolled?

Page 8 line 17. Can the authors justify why they are restricting to those who can ambulate to some degree? Non-ambulatory disease progression events continue to be relevant for wheelchair bound PwMS – i.e. loss of hand functioning, processing speeds, brain atrophy, QoL, etc.

Please define all abbreviations at first use (i.e T25FW), and there are v. minor grammatical errors (i.e. secundary, remittent vs remitting in Table 1) throughout.

Is there any information on race, ethnicity, or ancestral country/region of origin?

How is diet measured? Will other environmental/behavioral factors be measured, i.e. exposure to pesticides, vaping, exercise, UV, supplements?

With an emphasis on PRO and clinical outcomes/landmarks, 8-13% of registrants are missing these data at inclusion (Table 1). Is this missing at random, or possibly driven by patients with more severe disease declining such information, or might it be incomplete data collection at select centers? Can the authors comment?

Page 12 line 48. Why construct training and testing dataset that are deliberately unbalanced for recruiting center? The authors should consider balancing the participants from all centers in both the training and testing data sets, as each center may be tied to unmeasured

confounders, i.e. different prescribing patterns, race/ethnicity distributions, etc. The authors should then consider accounting for center differences in their models. Will any feature/variable selection strategies be employed to construct the prediction models, or will the authors rely on association testing etc?

The authors will be examining numerous relationships. They should acknowledge that they power calculation does not account for multiple testing. Furthermore, the power calculation seems incomplete: shouldn't the time frame, # of events be key parameters in the calculation?

The symptoms/measures that will be considered for progression analyses (pg10 line 36) can be enumerated.

Can the authors comment who they expect their findings to impact (who is the target population to which these findings will be generalizable to)?

VERSION 1 - AUTHOR RESPONSE

Reviewer: 1
Dr. Souraya El Sankari , Department of Neurology, Cliniques Universitaires Saint-Luc
Comments to the Author:
In this paper from the OFSEP group, the authors aim to collect broad multi parametric multi center determinants of progression in MS. The study design and objectives are provided. The studied parameters are specified with details. the manuscript is well written.

- Answer: We appreciate this comment.

A few questions / remarks for minor corrections:
1) Are authors intending to collect and use data from LP (OCB / free kappa light chains) for prognostic purposes ?

- Answer: We understand the potential interest to look at these biological markers in cerebrospinal fluid (CSF) beyond their diagnosis value (which was synthetized in recent reviews Mult Scler. 2023 Feb;29(2):169-181, PLoS One. 2019 Apr 15;14(4):e0215410.). CSF sampling was collected in clinical routine at the time of diagnosis with documentation of OCB and IgG index, and recorded in the OFSEP cohort database. It can be retrieved for this project. But we did not collect CSF in the biobank, therefore we don't have an analysis of free kappa light chains. Thank you for the suggestion, however.

- *Action: the manuscript has been completed (page 8)*

2) Combined to smoking, BMI, are vit D levels available in baseline evaluations of patients . the same for the EBV serological status

- Answer: as indicated in the method section (page 9), Vitamin D will be assessed from serum sample with mass spectrometry. While EBV serological status assessment was not planned, using the biobank collected will offer this opportunity, as mentioned in the method section (page 9) and we thank the reviewer for the suggestion.

- *Action: The text has been amended where required (page 9).*

3) Please check the citations style in the bibliography section. Many errors to be corrected, and the style to be checked.

- Answer: The bibliography has been checked

- *Action: Errors in the reference list have been corrected and the style harmonized.*

Reviewer: 2
Dr. Farren Briggs, University of Miami Miller School of Medicine
Comments to the Author:
This is an overview of a cohort study of MS patients recruited from 28 MS centers in France. The authors present an initial demographic/clinical description of subjects.

There are several opportunities to improve the document:

Page 7 line 20: The citations 16-18 refer to reviews of self-management interventions and exercise on QOL. They do not refer to registries or cohort that have successfully incorporated PROs, and leveraged them as outcomes or predictors (in cross-sectional and longitudinal analyses) in numerous studies.
For example: https://doi.org/10.7224/1537-2073.2020-133, https://doi.org/10.1016/j.msard.2019.01.045, https://doi.org/10.3389/fneur.2020.00632, etc. Thoughtful inclusion of appropriate citations would be an improvement.

- Answer: We appreciate the right notice and the proposed articles for citations that had escaped to our attention.

- *Action: We have replaced and quoted appropriate references (page 6).*

Page 7 Line 34. Please see https://doi.org/10.3389/fneur.2020.00632 - this ongoing study is very similar to what the authors describe in the opening of the paragraph.

- Answer: Indeed, this cohort study is similar to OFSEP, though it is not organized with as accurate and structured measurement times as in OFSEP-HD.

- *Action : the manuscript has been amended to quote this study (page 6).*

Page 8 Line 10, is there any information on enrolment rate of eligible PwMS from the 28 MS centers? Are the eligible patients at these 28 MS centers representative of the broader French MS population (i.e. are these centers only in urban centers?) In review of the CONSORT diagram (fig 1) – are there any concerns with selection bias, i.e. are the 30K at non-participating centers similar to the 41K at participating centers? Are the 16K who declined/not invited similar to the 2847 who enrolled?

- Answer: First, there was a typo as we have 25 – not 28 - MS centers in the cohort, which are located in urban centers but receiving MS people from larger area (general practitioners and neurologists from several counties referring to each center). Second, we compared the characteristics of 2847 included and 16239 non included MS individuals among 19806 eligible individuals, according to inclusion criteria, attending the 25 centers over the period. The data are presented in supplementary Table S1, and show that among eligible MS individuals, those included have less MRI activity in the past 3 months, a MS diagnosis for more than 6 months, a moderate EDSS, and receive more highly effective therapy, with a significant heterogeneity of

recruitment across MS centers. Such difference will have to be accounted for in the interpretation of the results.

- *Action: the text has been modified (page 11) and the comparison is provided in supplementary Table S2.*

Page 8 line 17. Can the authors justify why they are restricting to those who can ambulate to some degree? Non-ambulatory disease progression events continue to be relevant for wheelchair bound PwMS – i.e. loss of hand functioning, processing speeds, brain atrophy, QoL, etc.

- Answer: The reviewer is perfectly right that prognosis at later time after loss of ambulatory capacities remains of great importance. Our research question was primarily dictated by the need to identify the best therapeutic strategies to limit the progression of the disease and associated disability. While this is of utmost importance in MS people with EDSS $\leq$ 7.0 and applied in recent approach to start effective therapy (increasingly available and diversified) early, such therapies are less available / prescribed in non-ambulatory people. Secondarily, limited funded time for follow up led us to restrict the scope of patients included.

Please define all abbreviations at first use (i.e T25FW), and there are v. minor grammatical errors (i.e. secundary, remittent vs remitting in Table 1) throughout.

- Answer: We have checked and amended all where relevant.

- *Action: corrected. On request of the Editor, Table 1 has been moved to Table S1.*

Is there any information on race, ethnicity, or ancestral country/region of origin?

- Answer: The information on geographical area of origin is collected in the OFSEP-HD cohort. In France, the collection of ethnicity is difficult, and requires to obtain a waiver by law on data protection. The material collected in the biobank will be used to identify genetic ancestry of the patients.

- *Action: This is added in the method section (page 9).*

How is diet measured? Will other environmental/behavioral factors be measured, i.e. exposure to pesticides, vaping, exercise, UV, supplements?

- Answer: behavioral factors (smoking, alcohol) are collected in the OFSEP-HD cohort, as mentioned page 11. The other factors (diet, environment, other behaviours) are not measured.

With an emphasis on PRO and clinical outcomes/landmarks, 8-13% of registrants are missing these data at inclusion (Table 1). Is this missing at random, or possibly driven by patients with more severe disease declining such information, or might it be incomplete data collection at select centers? Can the authors comment?

- Answer: We thank the reviewer for paying attention to these points. A comparison of their characteristics at inclusion showed that individuals with missing data on clinical outcomes (22.4%), PROs (13.5%), and landmarks (19.2%), were older, with MS onset or MS diagnosed at older age, had slightly lower education, more progressive phenotype, higher EDSS score, and received less active treatment than those without missing data, independently of centers. Therefore, this was likely not missing at random.

- *Action: this is now reported in the results section (page 12), and in supplementary Tables S3-S5.*

Page 12 line 48. Why construct training and testing dataset that are deliberately unbalanced for recruiting center? The authors should consider balancing the participants from all centers in both the training and testing data sets, as each center may be tied to unmeasured confounders, i.e. different prescribing patterns, race/ethnicity distributions, etc. The authors should then consider accounting for center differences in their models. Will any feature/variable selection strategies be employed to construct the prediction models, or will the authors rely on association testing etc?

- Answer: The random selection of two thirds of the centers for training and remaining third for testing datasets was dictated by the fact that random choice at the individual level in the whole cohort had little chance to detect differences in performance when applied to sample with random differences, while the only consequence was in reducing the number in the testing set, ie the statistical power. Meanwhile, choosing different centers, at random, will precisely target measure and unmeasured confounders which provides more robust validity to the results (PMID: 27262237). External validation can be performed from patients who do not participate for learning and from alternative geographical areas and/or calendar periods. The random selection of the individuals would have been an internal validation strategy, while we aim to propose tools for medical decision making outside patients included in the OFSEP-HD. We proposed a random selection of centers for a geographical external validation of the predictive tools.

- *Action: Manuscript has been modified.*
  According to data from the remaining one third of centers in the external validation cohort, we will use several metrics to appraise the predictive capacities of the models. Note that we will randomize centers and not individuals for learning and validation, because the latter strategy would have been considered as internal validation, questioning the generalization of the results (PMID: 27262237) (page 12).

The authors will be examining numerous relationships. They should acknowledge that they power calculation does not account for multiple testing. Furthermore, the power calculation seems incomplete: shouldn't the time frame, # of events be key parameters in the calculation?

- Answer: We acknowledge that our power calculation does not account for multiple testing, as do other cohorts with many exploratory objectives. Furthermore, the procedure used for sample size calculation used proportion of events under the assumption of proportionality of risks, which is a way to account for number of events over a period of time, according to Hsieh et al (PMID: 11146149). There was a typo in the text where 'exposition rate' should read 'event rate'.

- *Action: This is now acknowledged. The typo has been corrected, and the method referenced (page 7).*

The symptoms/measures that will be considered for progression analyses (pg10 line 36) can be enumerated.

- Answer: we will consider neurological symptoms and signs which could be numerous, and tedious for a full enumeration.

- *Action: The text has been amended accordingly (page 9).*

Can the authors comment who they expect their findings to impact (who is the target population to which these findings will be generalizable to)?

- Answer: The target population is inferred from the inclusion criteria. To summarize this we can synthetize their characteristics as adult people with diagnosed MS with limited disability progression with preserved ambulatory ability, whatever the disease duration, potentially eligible to DMT, and followed in a French expert center, so representing a large proportion of French MS ambulatory people.

- *Action: This has been added at the end of the manuscript (page 13).*

Reviewer: 1
If you have selected 'Yes' above, please provide details of any competing interests.: not applicable

Reviewer: 2
If you have selected 'Yes' above, please provide details of any competing interests.: Not applicable

---

VERSION 2 - REVIEW

**Reviewer**	**2**
**Name**	**Briggs, Farren**
**Affiliation**	**University of Miami Miller School of Medicine**
**Date**	**19-Mar-2025**
**COI**	

The authors have thoughtfully responded to all concerns.
